# Microstructure-Dependent Visible-Light Driven Photoactivity of Sputtering-Assisted Synthesis of Sulfide-Based Visible-Light Sensitizer onto ZnO Nanorods

**DOI:** 10.3390/ma9121014

**Published:** 2016-12-15

**Authors:** Yuan-Chang Liang, Cheng-Chia Chung, Ya-Ju Lo, Chein-Chung Wang

**Affiliations:** Institute of Materials Engineering, National Taiwan Ocean University, Keelung 20224, Taiwan; johy53069@hotmail.com.tw (C.-C.C.); yalulo0807@gmail.com (Y.-J.L.); abc2589tw@gmail.com (C.-C.W.)

**Keywords:** sputtering, microstructures, heterostructure, photoactivity

## Abstract

The ZnO-CdS core-shell composite nanorods with CdS shell layer thicknesses of 5 and 20 nm were synthesized by combining the hydrothermal growth of ZnO nanorods with the sputtering thin-film deposition of CdS crystallites. The microstructures and optical properties of the ZnO-CdS nanorods were associated with the CdS shell layer thickness. A thicker CdS shell layer resulted in a rougher surface morphology, more crystal defects, and a broader optical absorbance edge in the ZnO-CdS rods. The ZnO-CdS (20 nm) nanorods thus engaged in more photoactivity in this study. When they were further subjected to a postannealing procedure in ambient Ar/H_2_, this resulted in the layer-like CdS shell layers being converted into the serrated CdS shell layers. By contrast, the ZnO-CdS nanorods conducted with the postannealing procedure exhibited superior photoactivity and photoelectrochemical performance; the substantial changes in the microstructures and optical properties of the composite nanorods following postannealing in this study might account for the observed results.

## 1. Introduction

Advancements have been substantial in the design and understanding of the physical properties of low-dimensional semiconductor heterostructures [1,2]. Low-dimensional semiconductor heterostructures, because of their special band alignment at the interfaces, have potential applications in photoactivated and gas-sensing devices [3,4]. Among various heterostructures consisting of two types of semiconductors, the type-II heterojunction provides an effective spatial separation of charge carriers upon photoexcitation by tuning the energy levels of the valence and conduction bands of the constituent semiconductors [5]. A wide-bandgap semiconductor is coupled with a narrow bandgap semiconductor to form a heterostructure, which extends the photoresponse into the visible-light region, after which enhanced light harvesting is expected [3]. Researchers have investigated numerous semiconductor heterostructure systems consisting of oxide and sulfide semiconductor—for examples, TiO_2_-CuS [6], ZnO/In_2_S_3_ [7], ZnO-CuS [8], ZnO-Ag_2_S [9], and ZnO-CdS [10,11]. ZnO, because of its high electron mobility and various fabrication methods, has garnered considerable attention for the fabrication and investigation of ZnO-based heterostructures for their enhanced light-driven properties [7,12]. The drawback of ZnO is its wide-bandgap value (approximately 3.3 eV), which induces the activation of ZnO only through UV irradiation. A visible-light sensitizer is therefore crucial for integration into ZnO to broaden the use of solar light when the device functions of ZnO-based materials necessitate operation under photoexcitation. 

Among various narrow-bandgap metal sulfide semiconductors, the CdS is a suitable visible-light sensitizer for ZnO, because CdS has a direct bandgap value in the visible-light region, and the crystallographic features of CdS are similar to those of ZnO; these advantages engender a close energy-band interaction between the two semiconductors when they are formed in a heterostructure [13,14]. An effective interband photoexcited charge transfer occurs between CdS and ZnO [14]. Several synthesis methods have been reported for fabricating CdS with various crystal features, including radio-frequency (RF) sputtering [15], successive ionic layer adsorption and reaction [16], the hydrothermal method [13], chemical bath deposition [17], thermal evaporation [18], chemical vapor deposition [19], and spray pyrolysis [20]. However, most ZnO-CdS heterostructure systems are synthesized by decorating CdS crystallites onto ZnO templates through aqueous chemical growth methods [10,11,14]. By comparison, RF sputtering is more advantageous for preparing CdS with various crystallite thicknesses and crystalline qualities. Incorporating CdS into the ZnO template is also advantageous for forming ZnO-CdS heterostructures through RF sputtering. The synthesis methods have a considerable influence on the microstructures of the semiconductors. Understanding the correlation between the crystal feature and photoactivated properties of the low-dimensional ZnO-CdS heterostructures by sputtering deposited CdS crystallites onto ZnO templates through a physical method is of scientific importance and relevant reports are limited in number. The sputtering-assisted synthesized ZnO-CdS heterostructures are favorable to investigate effects of structural modification of CdS crystallites on photoexcited properties of the heterostructures; this information is important for designing ZnO-CdS nanorods through a vacuum process with a satisfactory photoactivity. In the present study, CdS crystallites with various thicknesses were RF-sputtered onto ZnO rods to form ZnO-CdS core-shell heterostructure rods; moreover, a simple postannealing procedure was adopted to modulate the crystal properties of CdS shell layer. The microstructures and photoactivated properties of the sputtering technique facilitated the formation of ZnO-CdS heterostructure nanorods, which were investigated in this study. 

## 2. Materials and Methods 

In this study, ZnO-CdS core-shell nanorods with two different CdS shell layer thicknesses were fabricated by sputtering CdS thin films onto the surfaces of the hydrothermally derived ZnO nanorod templates (hereafter referred to as ZnO-CdS (5 nm) and ZnO-CdS (20 nm)). The CdS thin films were fabricated using RF magnetron sputtering in pure Ar ambient at 250 °C. The gas pressure during thin-film deposition was fixed at 2.67 Pa, and the sputtering power was fixed at 40 W. Detailed experiments on the synthesis of vertically aligned ZnO nanorods have been described elsewhere [21]. Some of the ZnO-CdS (20 nm) nanorods are subjected to a postannealing procedure in ambient Ar/H_2_ (3% H_2_ balanced) for 20 min at 400 °C to modulate crystal feature of the CdS crystallites.

Crystal structures of the as-synthesized core-shell rods were investigated by X-ray diffraction (XRD) using Cu Kα radiation. The surface morphologies of the various samples were characterized by scanning electron microscopy (SEM), and high-resolution transmission electron microscopy (HRTEM) was used to investigate the detailed microstructures of the core-shell rods. Room-temperature-dependent photoluminescence (PL) spectra were obtained using the 325 nm line of a He-Cd laser. The analysis of absorbance spectra of the core-shell rods were conducted by using UV-Vis spectrophotometer. Photocatalytic activity of various samples were performed by comparing the degradation of aqueous solution of methylene blue (MB; 10^−6^ M) with various core-shell nanorods as catalysts under visible light (λ > 420 nm) irradiation excited from the 100 W Xe arc lamp equipped with an ultraviolet light filter. The solution volume of MB is 30 mL and nanorods are grown on the pure glass substrates with a fixed coverage area of 2.0 cm × 2.0 cm for photocatalytic test use. The photoelectrochemical (PEC) properties were measured in a convenient three electrodes electrochemical system. A mixed aqueous solution composed of Na_2_S (0.25 M) and Na_2_SO_3_ (0.35 M) were used as electrolyte. Work electrodes were made of various core-shell nanorods on the conductive fluorine-doped tin oxide glasses. Ag/AgCl (1 M KCl) electrode was used as a reference electrode and a platinum wire was used as a counter electrode. The PEC measurements were conducted under visible light irradiation in this study.

## 3. Results and Discussion

**Figure 1:**
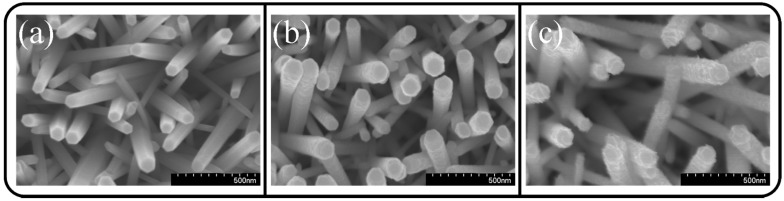
(**a**) SEM micrograph of ZnO-CdS (5 nm) nanorods; (**b**) SEM micrograph of ZnO-CdS (20 nm) nanorods; (**c**) SEM micrograph of ZnO-CdS nanorods annealed.

The surface morphologies of the various ZnO-CdS composite nanorods are shown in Figure 1a–c. Figure 1a displays the smooth surface of the ZnO-CdS (5 nm) nanorods. Moreover, the hexagonal morphology of the ZnO nanorods was maintained when the ultrathin 5-nm-thick CdS layer was coated onto the surfaces of the ZnO rods. By contrast, the ZnO nanorods coated with 20-nm-thick CdS shell layers exhibited increased surface roughness (Figure 1b); the rod surfaces changed to exhibit ruggedness. When the ZnO-CdS (20 nm) nanorods were further postannealed in ambient Ar/H_2_, the rod surfaces became relatively uneven. The surfaces of the composite rods were composed of many irregularly shaped, tiny island-like crystallites, according to an observation of the SEM image (Figure 1c).

**Figure 2:**
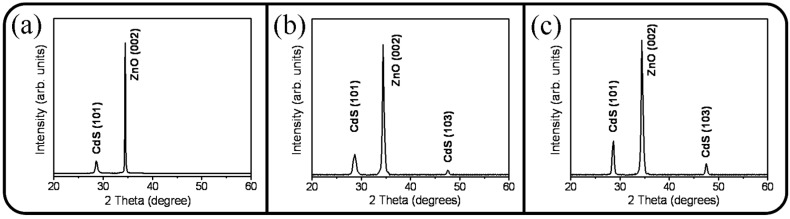
XRD patterns: (**a**) ZnO-CdS (5 nm) nanorods; (**b**) ZnO-CdS (20 nm) nanorods; (**c**) ZnO-CdS nanorods annealed.

Figure 2a–c display the XRD patterns of the ZnO-CdS nanorods with various CdS shell morphologies. The XRD patterns show two marked Bragg reflections centered at approximately 28.2° and 34.4° in Figure 2a; these reflections are ascribed to the Bragg reflections of the hexagonal CdS (101) (JCPDS No. 41-1049) and that of the hexagonal ZnO (002) (JCPDS No. 05-0664), respectively. The XRD results revealed that the ZnO crystals exhibited a high c-axis orientation, and the as-deposited CdS crystallites exhibited a strong (101)-crystallographic orientation. By contrast, a weak Bragg reflection centered at approximately 48.8° was observed for the thicker CdS shell layer in Figure 2b; this Bragg reflection was ascribed to the hexagonal CdS (103). When the CdS shell layer thickened, the crystallographic orientation of the CdS crystallites became more randomly oriented. The degree of preferred (101)-crystallographic orientation was evaluated by calculating the peak intensity ratio I_101_/(I_101_ + I_103_) and the background intensity was deducted before the calculation of peak intensity ratio. The peak intensity ratio was reduced to 78% for the thicker CdS shell layer. Furthermore, as displayed in Figure 2c, when the ZnO-CdS (20 nm) nanorods were further postannealed in ambient Ar/H_2_, the Bragg reflections of the CdS (101) and (103) became more sharp and intense; however, this annealing treatment further engendered an increased randomness of the crystallographic orientation of the 20 nm-thick CdS shell layer and the degree of preferred (101)-crystallographic orientation was decreased to 72%. The change of average crystallite size of thicker CdS shell layers with and without the postannealing procedure was evaluated from the XRD Bragg reflection of (101) according to Scherrer equation [22]. The average crystallite size of the CdS shell layer was increased approximately from 12.1 to 16.9 nm after the postannealing procedure. The XRD results revealed that the crystalline quality of the sputtering deposited CdS crystallites was markedly improved after the postannealing procedure. 

**Figure 3:**
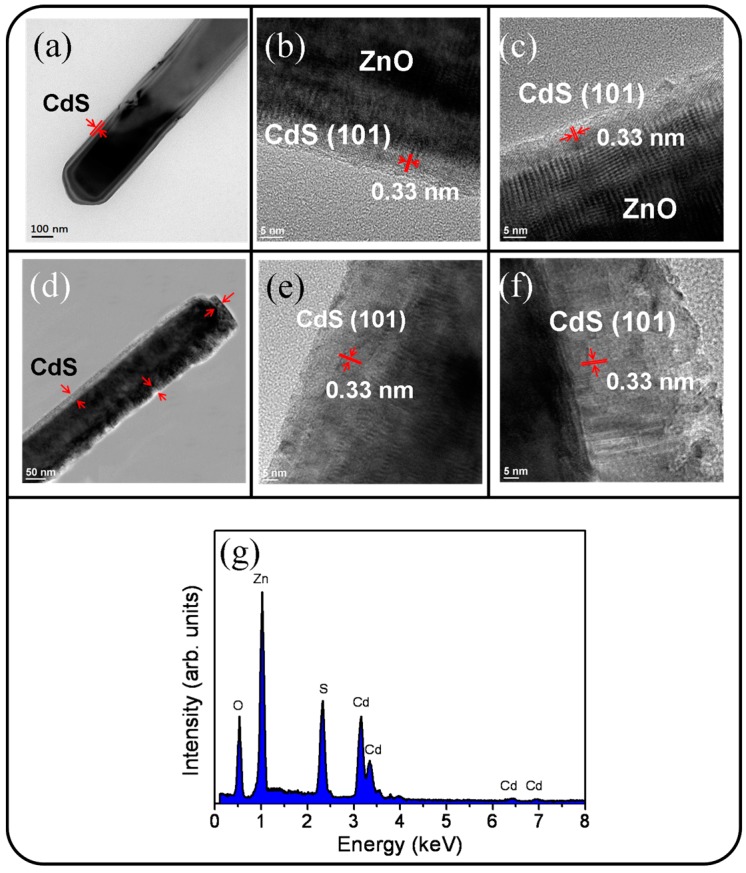
TEM analyses of the ZnO-CdS rods: (**a**) Low-magnification TEM image of the ZnO-CdS (5 nm) nanorod; (**b**,**c**) HRTEM images taken from the local regions of the nanorod; (**d**) low-magnification TEM image of the ZnO-CdS (20 nm) nanorod; (**e**,**f**) HRTEM images taken from the local regions of the nanorod; (**g**) EDS spectrum taken from the nanorod.

Figure 3a displays a low-magnification TEM image of the morphology of a single ZnO-CdS (5 nm) nanorod. The TEM image shows that the CdS crystallites were homogeneously coated onto the surface of the ZnO core to form a flat and continuous layer-like shell structure. The surface of the CdS shell layer was smooth. Figure 3b,c display high-resolution (HR) TEM images obtained from the edges of the ZnO-CdS nanorod. The clear and highly ordered lattice fringes with an interval of approximately 0.33 nm in the CdS shell layer corresponded to the (101) lattice plane. An ultrathin CdS shell layer with a high crystalline quality was formed on the surface of the ZnO core. Figure 3d displays a low-magnification TEM image of the morphology of a single ZnO-CdS (20 nm) nanorod. The thickness distribution of the CdS shell layer over the ZnO core was relatively inhomogeneous compared with that in the ZnO-CdS nanorod with a 5 nm-thick CdS shell layer. Figure 3e,f display the HRTEM images obtained from the outer regions of the ZnO-CdS nanorod. The ordered lattice fringes in the HRTEM images show that the excellent crystalline CdS shell layer covered the ZnO core. Certain portions of the CdS layer still had flat surfaces (Figure 3e). However, partial regions of the CdS shell layer exhibited irregular surfaces (Figure 3f); boundaries between the CdS crystallites were visible. The energy-dispersive X-ray spectroscopy (EDS) spectra displayed in Figure 3g show that Zn, Cd, O, and S are the main constituent elements of the selected composite rod. 

**Figure 4:**
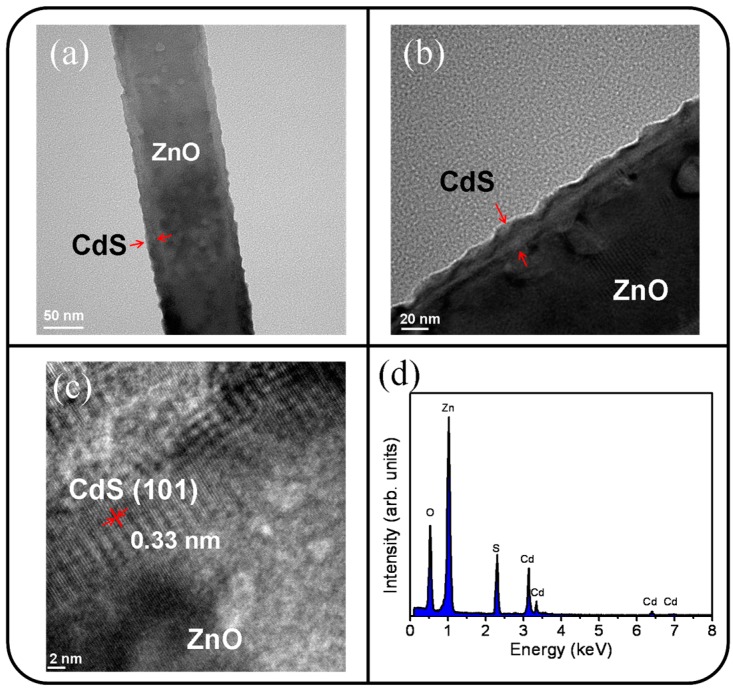
TEM analyses of the ZnO-CdS nanorod annealed: (**a**) Low-magnification TEM image; (**b**) high-magnification image; (**c**) HRTEM image taken from the local regions of the nanorod; (**d**) EDS spectrum taken from the nanorod.

Figure 4a displays the low-magnification TEM image of the ZnO-CdS nanorod conducted with a postannealing procedure. The image clearly shows that the surface morphology of the shell layer exhibited a serrated feature. The postannealing procedure engendered a morphological transformation of the as-deposited CdS crystallites and resulted in a layer-like CdS shell to convert into an undulated CdS shell which can be clearly observed in a high-magnification image in Figure 4b. The composite nanorod surface became rough after the postannealing treatment. Figure 4c displays the HRTEM image of the interfacial region of the ZnO-CdS nanorod. The ordered lattice fringes with an interval of approximately 0.33 nm and 0.26 nm corresponded to the lattice distance of the crystallographic planes of the CdS (101) and ZnO (002). The elemental composition of the selected composite rod was evaluated to be Zn, Cd, O, and S elements from the EDS spectra in Figure 4d.

**Figure 5:**
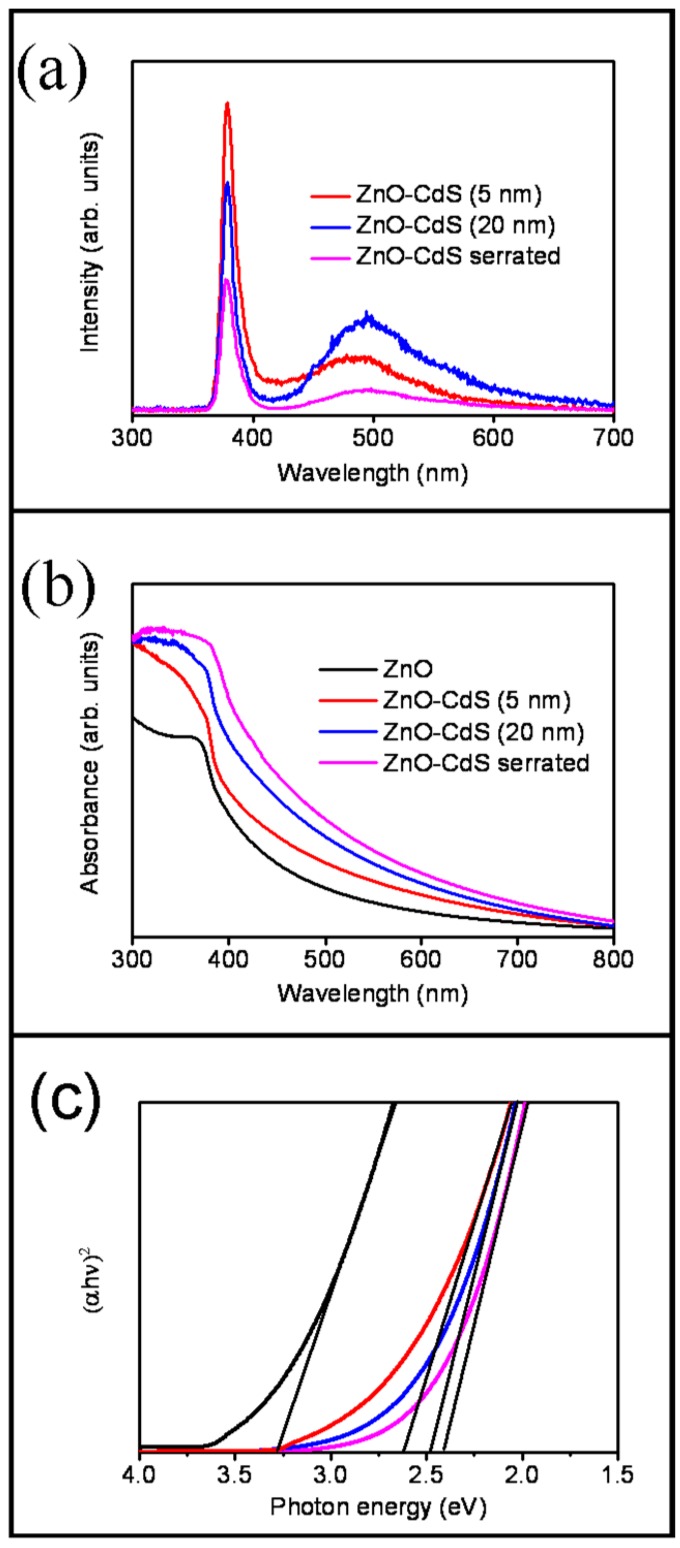
(**a**) PL(photoluminescence) spectra of the various ZnO-CdS nanorods; (**b**) optical absorbance spectra of the various ZnO-CdS nanorods; (**c**) Tauc plots of various nanorod samples for evaluating their optical bandgap values.

The room-temperature PL spectra of ZnO-CdS nanorods with various CdS shell layers are displayed in Figure 5a. A UV emission band centered at approximately 378 nm was ascribed to the near-band edge (NBE) emission of the ZnO nanorods [1,23]. Moreover, a clear visible-light emission band centered at approximately 495 nm was observed for the ZnO-CdS nanorods. For the ZnO-CdS composite nanorod system, this visible-light emission band is referred to as the deep-level or trap-state emission band, and is associated with structural defects arising from the sulfur-related point defects of the CdS shell layer [20,24]. A similar visible-light emission band at approximately 506 nm was also reported for a TiO_2_-CdS composite rod system [25]. Comparatively, the intensity of NBE emission band of ZnO core decreased and visible-light emission band intensity increased when the CdS shell thickness was increased. This is attributable to the factor that an increased size of surlfide surface defect density is highly associated with the thickness of CdS shell layer and a larger amount of CdS crystallites as a shell layer promoted the photoexcited charge separation efficiency of the ZnO core. The subsequently postannealing procedure for the ZnO-CdS nanorods in ambient Ar/H_2_ engendered the intensities of the ZnO core-dependent NBE band and visible-light emission band from the CdS shell quenched. The PL analysis result revealed that the charge separation efficiency of the ZnO core and the crystalline quality of the CdS shell were substantially improved when the ZnO-CdS core-shell nanorods were subjected to the postannealing procedure. Figure 5b displays the optical absorbance spectra of the ZnO-CdS nanorods with various CdS shell layers. By comparison, a clear redshift was observed for the ZnO nanorods coated with the CdS shell layers. This is because CdS crystallites have an optical bandgap value in the visible-light region (approximately 2.4 eV), which improved light harvesting to the solar lights of the wide-bandgap ZnO [26]. Furthermore, the thicker 20-nm-thick CdS shell layer engendered the ZnO-CdS nanorods with a larger redshift and a broader optical absorbance edge in comparison with the ZnO-CdS nanorods containing a 5-nm-thick shell layer. Thicker CdS crystallites have a rougher surface; this broadened the optical absorbance edge of ZnO-CdS with a thicker CdS shell layer [4]. The broadness of optical absorbance edge of the ZnO-CdS nanorods was further widened when the layer-like CdS shell was converted into the serrated CdS shell. This might be attributed to the serrated CdS shell layer is more favorable for light harvesting because of multiple light reflections and scattering [4]. The corresponding optical bandgap values are evaluated and exhibited in Figure 5c. The optical bandgap values of the ZnO-CdS (5 nm), ZnO-CdS (20 nm), and ZnO-CdS nanorods with the postannealing procedure are approximately 2.61, 2.48, and 2.41 eV, respectively. The ZnO-CdS nanorods with the postannealing procedure exhibited an enhanced light harvesting ability among the samples.

**Figure 6:**
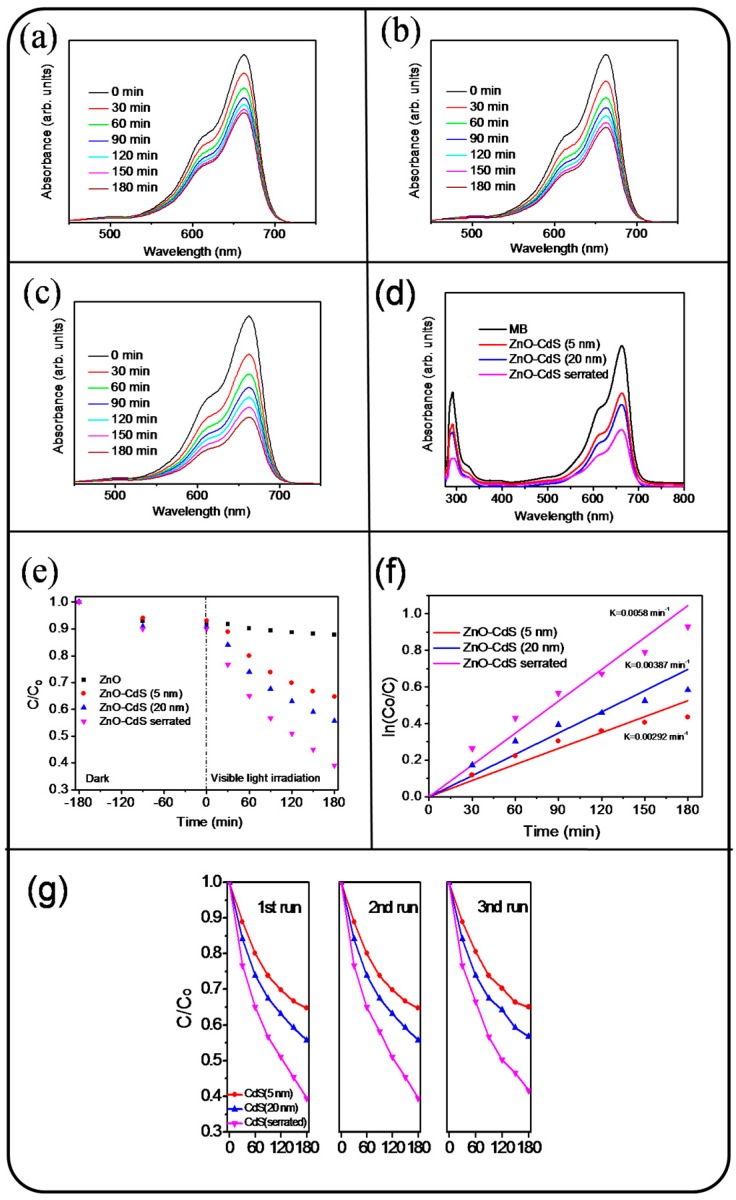
Intensity variation of absorbance spectra of MB solution vs. degradation duration containing various nanorod samples under visible-light illumination: (**a**) ZnO-CdS (5 nm) nanorods; (**b**) ZnO-CdS (20 nm) nanorods; (**c**) ZnO-CdS nanorods annealed; (**d**) intensity variation of absorbance spectra of MB solution containing various rod samples after 180 min visible-light illumination in the wavelength range from UV to visible region; (**e**) C/C_o_ vs. irradiation time curves for MB solution with various nanorods samples in dark conditions and under visible-light illumination; (**f**) plot of ln(C_o_/C) vs. reaction time for MB solution containing various nanorod samples under visible-light illumination; (**g**) recycled performances (three test runs) in the presence of various ZnO-CdS nanorods for photodegradation of MB dye.

The photocatalytic performance of the ZnO and ZnO-CdS nanorods with various CdS shell layers under visible-light irradiation was further investigated. The time-course photodegradation curves for an aqueous MB solution with various rods samples are displayed in Figure 6a–c. The visible and intense peaks of the absorbance spectra at approximately 663 nm were due to monomeric MB. The ratio of the remaining MB concentration (C) after visible-light irradiation to the initial MB concentration without visible-light irradiation (C_o_) i.e., C/C_o_ was used to determine the photodegradation degree of the MB solution containing various rod samples. Therefore, the C/C_o_ value could be evaluated from the absorbance spectra intensity ratio at 663 nm before and after the MB solution was subjected to visible-light irradiation with various nanorod samples. Figure 6d shows the absorbance spectra of the MB solution containing various rod samples at the irradiation duration of 180 min in the wavelength range from UV to visible region. The decrease in the absorbance peak intensity in the UV region is associated with the mineralization of the MB dye during the photodegradation process. To evaluate the influence of MB amount adsorbed on photocatalytic activity, dark adsorption tests were performed at different durations (90 and 180 min). Figure 6e displays the C/C_o_ versus irradiation duration for the MB solution with various nanorod samples. The slight MB concentration changes in dark conditions are influenced by the adsorption of MB on surfaces of various nanorod samples. The concentration of the MB solution containing pure ZnO nanorods as the photocatalyst remained nearly unchanged under visible-light irradiation at different durations. By contrast, the coating of the CdS shell layers onto the ZnO nanorods improved photodegradation efficiency. Pure ZnO nanorods, because of their large bandgap, did not cause a substantial photodegradation reaction under visible-light irradiation, whereas the ZnO nanorods coated with CdS crystallites, which have the bandgap value in the visible-light region, produced photoexcited carriers at the shell layers. The CdS shell layer under visible-light irradiation induced photoexcited electron transportation across the heterointerfaces of ZnO/CdS [27]. The prolonged lift time of the photoexcited electron-holes, because of the presence of heterointerfaces, improved the production efficiency of surface hydroxyl groups in photodegradation tests in the MB solution containing ZnO-CdS nanorods under visible-light irradiation. For a quantitative comparison of the photocatalytic activities of various rods samples, the reaction rate constant was evaluated by adopting the pseudo-first-order model from the plot of ln(C_o_/C) versus reaction time (Figure 6f) [28]. The reaction rate constants were determined to be approximately 0.0029, 0.0039, and 0.0058 min^−1^ for the MB solution in the presence of ZnO-CdS (5 nm), ZnO-CdS (20 nm), and ZnO-CdS nanorods with the postannealing procedure, respectively, under visible-light irradiation. The ZnO-CdS nanorods with a thicker CdS shell layer exhibited a photodegradation performance superior to that of the rods with a thinner CdS shell layer. Moreover, the ZnO-CdS nanorods with the postannealing procedure exhibited the best photodegradation performance among the various rod samples. According to the mentioned microstructural analysis, the ZnO-CdS nanorods exhibited serrated CdS shell layers, which rendered relatively rugged surfaces for the ZnO-CdS nanorods. Furthermore, a higher degree of crystalline quality in CdS crystallites and a broader optical absorbance edge of the ZnO-CdS nanorods among the various samples might account for their superior photocatalytic activity under visible-light irradiation. To confirm the recycling ability and long-term chemical stability of the ZnO-CdS nanorods, the degradation test of the MB solution containing ZnO-CdS nanorods under visible-light irradiation was repeated three times (Figure 6g). From Figure 6g, the C/C_o_ values of the ZnO-CdS (5 nm), ZnO-CdS (20 nm), and ZnO-CdS nanorods with the postannealing procedure after 180 min visible-light irradiation are approximately 0.65, 0.57, and 0.4 in the first cycle test. Moreover, those values are 0.67, 0.58, and 0.41 in the third cycle test under the same measurement condition. No significant variation was found in the photodegradation efficiency of the various ZnO-CdS nanorods; moreover, the ZnO-CdS nanorod catalyst conducted with the postannealing procedure in ambient Ar/H_2_ was stable and reusable for degrading MB dye with a relatively high visible-light driven photodegradation performance among the various nanorod samples.

**Figure 7:**
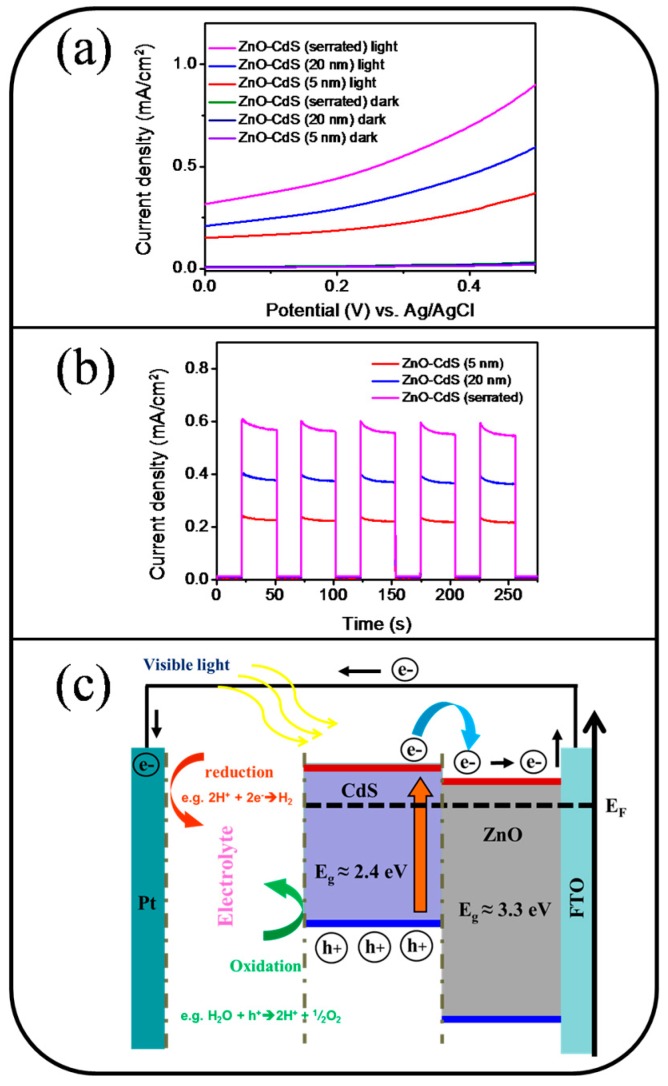
(**a**) Current density vs. potential curves for various nanorod samples with and without visible-light illumination; (**b**) cyclic current density vs. time curves for various nanorod samples under chopped visible-light illumination; (**c**) illustration of band alignment and charges transfer of the ZnO-CdS heterostructure under visible-light illumination.

Figure 7a displays the current density versus potential curves for the various nanorod samples in the presence and absence of visible-light irradiation during PEC measurements. The measured current density of all nanorod samples in the absence of visible-light illumination was relatively small over the swept voltage ranges. By contrast, the measured current density of the various ZnO-CdS nanorod samples markedly increased upon exposure to visible light, indicating enhanced visible-light harvesting by a shell architecture. Figure 7b displays the cyclic photocurrent density versus the time curves of the various rod samples obtained at 0.3 V under chopped visible-light irradiation. The high repeatability of the photocurrent density versus time curves of the various samples revealed that the as-synthesized ZnO-CdS nanorods were chemically stable for use as photoanodes in this study (Figure 7b). The photocurrent densities of the ZnO-CdS (5 nm), ZnO-CdS (20 nm), and ZnO-CdS nanorods conducted with the postannealing procedure at 0.3 V were approximately 0.23, 0.38, and 0.58 mA/cm^2^, respectively, under visible-light illumination. The reduction-oxidation reactions in the electrolyte during the PEC measurements involved the electron-hole pairs that presented in the CdS shell layers upon exposure to visible-light irradiation (Figure 7c). The photoexcited holes remained in the valence band of the CdS; however, the CdS shell layers inject the photoexcited electrons from its conduction band into that of the ZnO nanorods. The injected electrons flowed through the ZnO nanorods, and reached the F-doped tin oxide layer [29]. The band alignment between the ZnO-CdS in this study was similar to that of the TiO_2_-CdS and TiO_2_-CdSe heterostructure systems, and these heterostructures have exhibited improved charge injection efficiency and decreased charge recombination loss compared with the constituent single counterpart under light irradiation [30]. For the ZnO-CdS nanorod system in this study, the composite rods with a thicker CdS shell layer exhibited superior PEC performance. This is associated with the composite rods with a thicker CdS shell layer being larger in visible-light harvesting; moreover, as the shell layer, larger CdS crystallites might provide a higher number of photoexcited carriers under light irradiation [31]. These factors enabled the ZnO-CdS nanorods with a thicker CdS shell layer to exhibit a higher photocurrent density compared with those with a thinner CdS shell layer. A similar shell layer crystallite-size effect on the photocurrent density was reported in oxide composite rods with two substantially different bandgap values [4]. By contrast, the ZnO-CdS nanorods conducted with the postannealing procedure exhibited a superior PEC performance compared with the ZnO-CdS nanorods without the postannealing procedure under the same effective CdS shell layer thickness. Based on the aforementioned structural analyses, this is attributable to the higher efficiency in visible-light harvesting in ZnO-CdS nanorods associated with the substantial microstructure and crystalline quality differences among the nanorod samples. 

## 4. Conclusions

For this study, the ZnO-CdS core-shell composite nanorods were synthesized by sputtering CdS crystallites onto hydrothermally derived ZnO rods. The ZnO-CdS composite nanorods with two CdS shell thicknesses of approximately 5 and 20 nm were fabricated. Microstructural analyses revealed that the CdS crystallites covered the surfaces of the ZnO nanorods homogeneously and formed a layer-like structure. The ZnO-CdS (20 nm) composite nanorods exhibited a rougher surface and more surface crystal defects compared with the ZnO-CdS (5 nm) nanorods. These differences engendered the ZnO-CdS (20 nm) nanorods with higher photoactivity and PEC performance. Further postannealing the ZnO-CdS (20 nm) nanorods in ambient Ar/H_2_ engendered the morphology change of the layer-like CdS shell to a serrated CdS shell layer. In this study, the photoactivity and PEC performance of the ZnO-based nanorods was further enhanced through a postannealing procedure, because of improvements in visible-light harvesting and crystalline quality of the CdS shell layer in the ZnO-CdS nanorods.
